# Response of Leafhopper Community Structure and Diversity to Fragmented Habitat in a Rocky Karst Desertification Area, Guizhou, China

**DOI:** 10.3390/insects17010042

**Published:** 2025-12-29

**Authors:** Wenming Xu, Jinqiu Wang, Yuanqi Zhao, Yuehua Song

**Affiliations:** 1 School of Karst Science, Guizhou Normal University, Guiyang 550025, China; 232100170559@gznu.edu.cn (W.X.); wjq1zb@163.com (J.W.); zyq19991208@163.com (Y.Z.); 2 State Engineering Technology Institute for Karst Desertification Control, Guiyang 550025, China

**Keywords:** leafhopper, habitat fragmentation, community structure, species diversity, genetic diversity

## Abstract

Guizhou Province is located in the karst area of southern China, where rocky desertification is severe, and habitat fragmentation is prominent. Leafhoppers are widely distributed, with over 2000 recognized species found in China. However, the impact of habitat fragmentation in karst areas on the biodiversity of leafhoppers has not been comprehensively explored. Leafhopper specimens were collected in Bijie City, Guizhou Province (a light to moderate karst rocky desertification area) from 2019 to 2022. Using methods such as the generalized linear model (GLM) and redundancy analysis (RDA), this study explored the impacts of habitat fragmentation and environmental factors (plants, soil, climate) on the genus-level and genetic diversity of leafhoppers. When the degree of habitat fragmentation decreased, the genus diversity of leafhoppers increased significantly. The GLM showed that among the characteristics of fragmented habitats, patch area had the greatest impact on the generic richness of leafhoppers. RDA indicated that gene exchange among leafhoppers was more frequent between patches with irregular boundaries, and leafhoppers in patches with small areas and complex boundaries had greater genetic diversity. As phytophagous insects, leafhoppers were mainly affected by host plants and less affected by soil properties. Mitigating habitat fragmentation had a positive impact on the biodiversity of leafhoppers, which emphasizes the necessity of protecting biodiversity by reducing habitat fragmentation in the future.

## Simple Summary

Guizhou Province’s karst areas display severe rocky desertification and prominent habitat fragmentation. This study focuses on the effects of habitat fragmentation and environmental factors (plants, soil, climate) on the genus-level and genetic diversity of leafhoppers in Bijie, Guizhou (an area with light to moderate rocky desertification). Habitat fragmentation had a negative impact on the generic diversity of leafhoppers, and among the characteristics of fragmented habitats, patch area exerted the greatest influence on leafhopper generic richness. Gene exchange among leafhoppers was more frequent between patches with irregular boundaries. As phytophagous insects, leafhoppers were mainly influenced by host plants and less affected by soil. Mitigating habitat fragmentation had a positive effect on the biodiversity of leafhoppers, which highlights the necessity of protecting biodiversity by reducing the rate of habitat fragmentation in the future.

## 1. Introduction

China is the country where karst landforms are most widely distributed. Guizhou Province (ranging from 103°36′ E to 109°35′ E in longitude and from 24°37′ N to 29°13′ N in latitude) is situated in the heart of the South China Karst region, with complex topography and distinctive environmental features [1,2]. Soil erosion and rocky desertification are the primary factors that impact the ecological environment of the karst ecosystem. They are also the main causes of the decline in biodiversity [3]. Due to soil erosion and a decrease in vegetation, the rocky desertification frequently destroys the ecological habitat [4,5]. Habitat fragmentation by the division and breaking of a once continuous environment into isolated, dispersed habitat patches, and the segmentation and isolation of biological populations in the area, thereby causing the loss of diversity at the levels of genes, species, and ecosystems [6,7,8]. In karst areas, rocky desertification and habitat fragmentation can cause double pressure on ecosystems and aggravate the loss of biodiversity [9,10,11,12]. Therefore, exploring the interaction between species diversity and ecosystems in karst areas is essential to preserving biodiversity.

Leafhoppers (Hemiptera: Cicadomorpha: Cicadellidae) are globally distributed and encompass approximately 2550 genera, with more than 21,000 species, including almost 2000 species in China [13,14,15]. Leafhoppers are prevalent pests in agricultural and forestry ecosystems, with most species feeding on plant sap as their primary nutritional source, and some specializing in leaf mesophyll cell contents—e.g., Typhlocybinae [16,17,18]. Leafhoppers are characterized by their small size, typically measuring between 3 and 15 mm in body length, and their migratory ability. Their habitat primarily includes forests and grasslands, with some species exhibiting a high degree of polyphagy, while others specialize exclusively in only one or a few plant species [19,20]. As an important part of the ecosystem, their population dynamics and distribution patterns are extremely sensitive to changes in environmental factors and can quickly and effectively reflect changes in the local ecological conditions [21,22]. The population dynamics and distribution pattern of leafhoppers are closely related to the natural environment, such as meteorological and vegetation conditions [23,24]. In this study, the term “leafhoppers” specifically refers to insects of the family Cicadellidae (order Hemiptera), and does not include froghoppers (family Cercopidae), planthoppers (superfamily Fulgoroidea), delphacids (family Delphacidae), or other related insect groups.

In this research, the study area of Bijie Salaxi, which represents a karst region experiencing light to moderate rocky desertification in Guizhou, was chosen for the collection of leafhopper specimens. First, combining with meteorological factors, we investigated the structure, diversity, and distribution pattern of leafhopper populations from the perspective of temporal dynamics. We aimed to explore the relationship between habitat fragmentation and the structure and diversity of leafhopper communities in different years and months in a rocky karst desertification area. Secondly, the relationship between genera diversity of leafhoppers and environmental factors such as plants and soil under fragmented patches was discussed. This study aimed to explore the theory of habitat fragmentation within karst rocky desertification habitats, specifically examining the relationship between genus richness and habitat patch area, and to propose relevant suggestions for reducing fragmentation and ecological restoration. In particular, we addressed the following hypotheses:

(i) A reduction in the degree of habitat fragmentation is conducive to increasing the diversity of leafhoppers at the genus level.
(ii) Habitat patches that are smaller in area or more isolated have fewer leafhopper genera.
(iii) The plant species richness on habitat patches has a positive impact on leafhopper community diversity.

## 2. Materials and Methods

### 2.1. Study Sites

The Bijie Salaxi Demonstration Zone (105°01′11″–105°08′38″ E, 27°11′09″–27°17′28″ N) is located in the Liuchong River basin of Qixingguan District in the northwest of Guizhou Province. The elevation ranges from approximately 1500 to 2200 m, with an average annual temperature of about 13 °C. Precipitation is mainly concentrated from July to September, characterized by a north subtropical humid monsoon climate [25]. The karst region within the Bijie Salaxi Demonstration Zone encompasses a significant expanse of light rocky desertification [26]. Karst landforms are widely distributed in the region, and habitat fragmentation is prominent. In addition, unreasonable exploitative practices in agricultural production have caused severe habitat destruction [27].

### 2.2. Sample Plot Setting and Sampling

**Figure 1:**
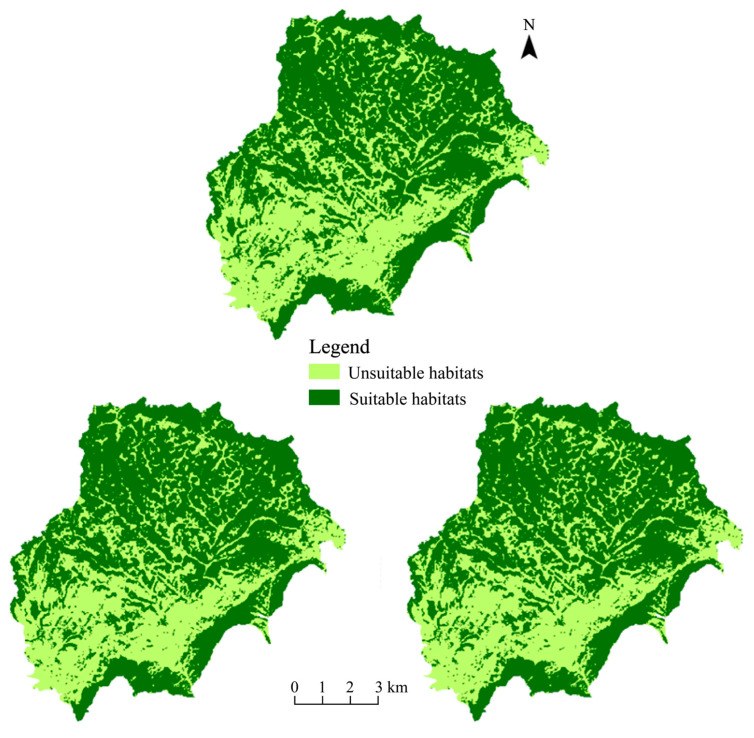
Changes in suitable habitats of leafhoppers in the study area from 2019 to 2021.

The suitable habitat of leafhoppers in the study area is not continuous, but presents a fragmented distribution pattern, indicating that the suitable living environment of leafhoppers is divided into several relatively isolated patches. In the south and northeast, vegetation types such as forests, shrubs, and grasslands are more conducive to the growth and reproduction of leafhoppers, so these places form large areas of suitable habitat. The more densely settled central and western regions are less suitable for leafhoppers (Figure 1).

**Figure 2:**
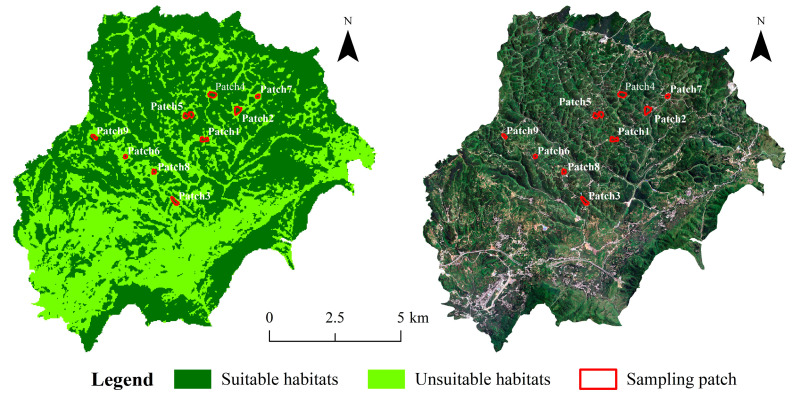
Distribution of sampling sites in the study area in 2022.

Based on the leafhoppers’ life history traits, occurrence patterns, and the study area’s vegetation coverage, nine patches were initially selected, with each patch including woodland, shrubland, and grassland. From May to October each year from 2019 to 2021, leafhoppers were collected, which indicated that May and August were the low and high abundance periods of leafhoppers in the Bijie area, respectively [12,28]. Therefore, we re-selected nine patches with different areas and perimeters, and three 200 m^2^ sample plots were established within each re-selected patch, resulting in a total of 27 sample plots, and conducted a more comprehensive survey in May and August 2022 (Figure 2).

Leafhopper specimens were collected on sunny days at the end of each month, during the warmer period of the day (9:00 to 18:00). Specimens were collected using a sweep net with a diameter of 30 cm and a depth of 50 cm. During the collection process, the net was swept back and forth along the two diagonals of each sample plot, for a total of 200 sweeps. For every set of 20 sweeps, the leafhoppers were collected into a centrifuge tube containing absolute ethanol, including dead or inactive leafhoppers from the bottom of the net. The meteorological data were obtained from the Salaxi meteorological station in Qixingguan District, Bijie City, Guizhou Province (105°2′18′′ E, 27°12′5′′ N). In May and August 2022, plant species composition was investigated at leafhopper sampling sites. In August 2023, the soil physical properties (water content, bulk density and total soil porosity) of 0–15 cm layer were measured by soil core method at each sampling point, and the chemical properties (total nitrogen, total carbon, organic matter, total phosphorus, pH, exchangeable calcium, exchangeable magnesium and electrical conductivity) were measured after the soil samples were brought back to the laboratory [29,30].

### 2.3. Species Identification and Gene Determination

In the laboratory, leafhopper specimens were identified to genus level. The morphology and genital characteristics of male leafhopper specimens were observed under an Olympus SZX16 stereomicroscope (Olympus Corporation, Tokyo, Japan). Female specimens were assigned to the same genus if their external morphological characteristics were consistent with those of the identified males collected from the same patch and during the same time period. In the identification and statistics of leafhoppers, only adult females and males were included, while nymphs were not. The species identification work was based on the 3I Word Auchenorrhyncha Databases (https://hoppers.speciesfile.org, accessed on 1 September 2022) and the leafhopper taxonomy-related literature [31,32,33]. The same leafhopper species were not widely distributed in every sample point and patch in the study area, which did not meet the sequencing requirements. Therefore, two common leafhopper species, *Evacanthus acuminatus* (Fabricius, 1794) and *Macrosteles fascifrons* (Stål, 1858), were selected from three patches (Patch 1, Patch 4, Patch 9) with large area differences, and their mitochondrial gene sequences (COI, Cytb, 16S) and two nuclear gene sequences (ITS2, 28S) were determined. MEGA 7.0 was used to align the sequenced leafhopper gene sequences [34]. Dnasp 5.0 was used to calculate haplotype numbers (h), haplotype diversity (Hd), nucleotide diversity (Pi), average number of nucleotide differences (K), and number of polymorphic sites (S) of leafhopper genes in different patches to analyze the genetic diversity of leafhoppers. The fixation index (Fst) and gene flow (Nm) of the two leafhoppers in different patches were calculated [35].

### 2.4. Data Statistics and Analysis

The habitat distribution map of leafhoppers in different years was drawn, and patch characteristics (patch area (pArea), patch perimeter (pPer), area perimeter ratio (pShp), and distance to the nearest suitable patch (DNSP)) were counted in Arcgis 10.2 [36], and the satellite images were derived from the geospatial data cloud (https://www.gscloud.cn/, accessed on 1 May 2022). Forests, shrubs, and grasslands are suitable habitats for leafhoppers, while bare cultivated land during the fallow period and other types of land are not [12]. Five habitat fragmentation landscape indices (patch density (PD), edge density (ED), contagion index (CONTAG), division index (DIVISION), and aggregation index (AI)) of the study area from 2019 to 2021 were calculated in FRAGSTATS 4.2 [37]. Excel 2016 and PAST 3 were used to conduct statistical analysis [38,39]. The number of leafhopper genera and individuals was counted, and their relative abundance was calculated. Leafhopper genera whose individual counts accounted for more than 5% of the total leafhopper individuals were defined as dominant; those accounting for 1% to 5% were defined as common; those accounting for less than 1% were defined as rare [40]. We also calculated the Shannon–Wiener diversity index, Margalef richness index, and Pielou evenness index [41,42,43,44].

Spearman correlation analysis was used to analyze the correlation between leafhoppers and meteorological data in SPSS 26 [45]. In order to explore the effects of patch area, perimeter, and area perimeter ratio on the genetic diversity of leafhopper genes, the genetic diversity index of Patch1, Patch4, and Patch9 leafhoppers and the patch characteristic index of the corresponding patches were analyzed using redundancy in Canoco 5 [46]. Pearson correlation analysis was used to analyze plant species composition and leafhopper genus diversity parameters to draw related scatter plots and radar charts. Generalized linear models were constructed in R4.3.2 using the ‘MuMIn’ package. The model.avg function was used to calculate the AICc of small samples to evaluate the optimal model. The model with the smallest AICc value was regarded as the optimal model, where ΔAIC (ΔAIC = AICi−AICmin).

## 3. Results

### 3.1. Leafhopper Community Structure and Genetic Diversity

#### 3.1.1. Composition and Diversity of Leafhopper Community in Different Years

A total of 36,681 leafhopper specimens were collected in the study area, belonging to 16 subfamilies and 109 genera. A total of 8444 (14 subfamilies, 77 genera), 9131 (15 subfamilies, 80 genera), and 9399 (15 subfamilies, 83 genera) specimens were collected from the nine sampling sites in May–October 2019, 2020, and 2021, respectively. In 2019, the dominant genera were Evacanthus, Paralaevicephalus, Dikraneura, Empoasca, and Empoascanara, with a combined total of 6099 individuals, accounting for 72.23% of the total number collected. Four genera, *Evacanthus*, *Macrosteles*, *Empoasca*, and *Empoascanara*, were dominant in 2020, and the number of individuals in these was 5438 (59.56%). *Evacanthus*, *Macrosteles*, *Dikraneura*, and *Empoasca* were the dominant genera in 2021, 4995 individuals (52.72%). In May and August 2022, 9707 (15 subfamilies, 81 genera) leafhopper specimens were collected from 27 sampling sites in nine patches, with Evacanthus, Macrosteles, Dikraneura, and Empoasca as the dominant genera. *Wyushinamia*, *Macrosteles*, *Exitianus*, and *Dikraneura* were the dominant genera of leafhoppers in 2022, 3334 individuals (34.3%) (Table 1).

The Shannon–Wiener index (H’) of leafhoppers from 2019 to 2022 showed an increasing trend. The Pielou evenness index also showed an upward trend, suggesting that the distribution of the number of individuals of each genus within the leafhopper community was becoming more even, and the community structure was becoming more stable. The Simpson index (C), which is negatively correlated with species diversity, decreased year by year, further confirming the continuous increase in the generic diversity of the leafhopper community. The Margalef richness index (R) first increased and then decreased, possibly due to the fact that leafhopper data were collected over six months in each of the previous three years, whereas only data from May and August were collected in 2022.

#### 3.1.2. Composition and Diversity of Leafhopper Communities in Different Patches

There were significant differences in the number of individuals and dominance of nine patches in May and August of 2022 (Table 2). Patch 2 had the most leafhopper subfamilies, with 11 subfamilies, while Patch 3 had only 8 subfamilies. Patch 2 and Patch 1 had the highest number of leafhopper genera, 46 genera, while Patch 7 had only 29 genera. Patch 3 had the largest number of individuals, which was 2051, while Patch 7 had the smallest number of individuals, which was 302. Six genera, such as *Empoascanara*, *Wyushinamia,* and *Handianus* became the dominant genera of Patch 3, and the number of individuals in these dominant genera was 1,725, which was the highest among all patches. The minimum number of individuals in the dominant genus of Patch 7 was 221, and five genera, including *Evacanthus*, *Macrosteles*, and *Hecalus*, were dominant. It is worth noting that Patch 7 had the smallest area, the fewest number of leafhopper individuals collected, and the lowest number of leafhopper genera.

**Figure 3:**
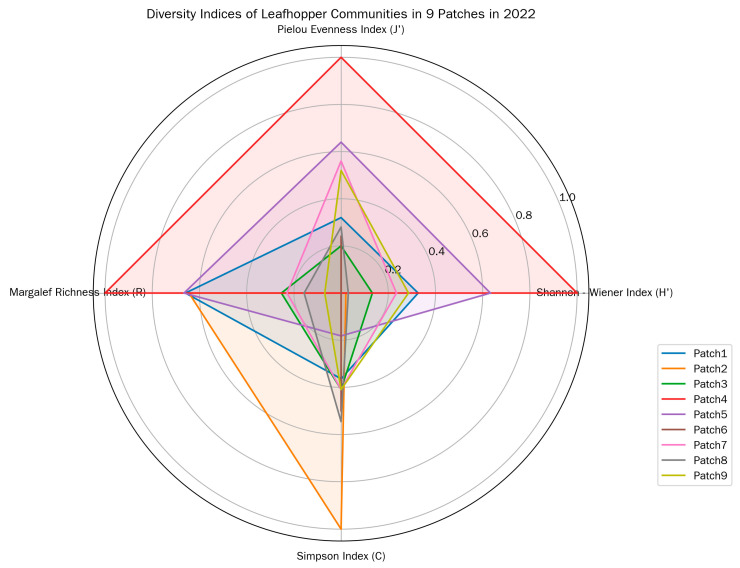
Diversity indices of leafhopper communities in different patches in 2022.

Patch 4 exhibited the highest leafhopper generic diversity, as reflected by its highest Shannon–Wiener diversity index (H′), Pielou evenness index (J′), and Margalef genera richness index (R); the even distribution of leafhopper individuals in this patch was also supported by its high J′. In contrast, Patch 2 and Patch 6 showed relatively low generic diversity, with lower values for these diversity and evenness indices—particularly Patch 2, where the uneven distribution of leafhopper genera was more pronounced (Figure 3).

Patch 4 also had the lowest Simpson dominance index (C), which further confirmed its high generic diversity, while Patch 2 had the highest C, consistent with its relatively poor diversity performance. In summary, Patch 4 and Patch 5 outperformed other patches in terms of leafhopper generic diversity, evenness, and richness, whereas Patch 2 performed relatively poorly. Notably, Patch 2 had a relatively large area (second only to Patch 5), suggesting that patch size alone does not fully determine leafhopper diversity.

Plant species number also varied across patches: Patch 4 had the highest number of plant species, while Patch 6 had the lowest—an observation that might link plant resource availability to leafhopper community structure.

#### 3.1.3. Genetic Diversity of Leafhoppers in Three Different Patches

There are significant differences in population genetic diversity between *E. acuminatus* and *M. fascifrons* in different patches (Table 3). Based on the combined analysis of mitochondrial gene and nuclear gene, the nucleotide diversity (Pi) of *E. acuminatus* and *M. fascifrons* were ranked as follows: Patch 9 (0.04921, 0.09253) > Patch 1 (0.04592, 0.09208) > Patch 4 (0.03560, 0.02325), respectively. The nucleotide diversity (Pi), average number of nucleotide differences (K), and number of polymorphic sites (S) of *M. fascifrons* among different patches were consistent with those of *E. acuminatus*. Among the three patches, Patch 9, which has the smallest area and the highest perimeter-to-area ratio, exhibits relatively high genetic diversity.

**Figure 4:**
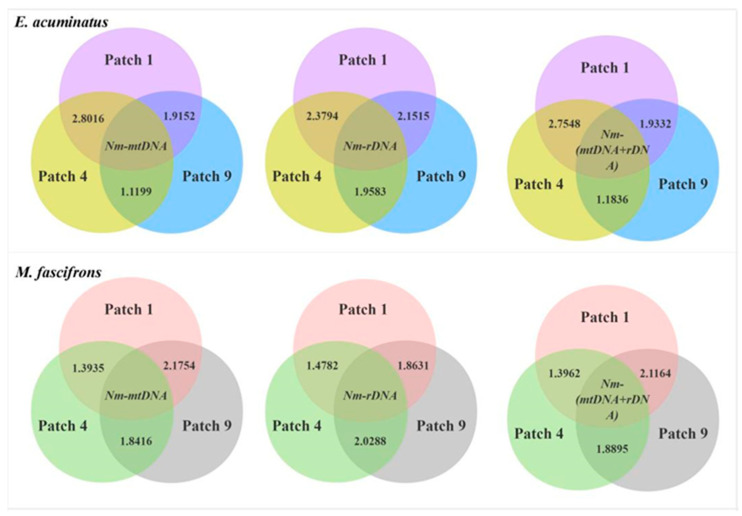
Gene flow (Nm) of leafhoppers in different patch populations calculated based on different genes.

The Nm values of gene flow between different patches for both *E. acuminatus* and *M. fascifrons* ranged from 1.1199 to 2.8016, indicating that there was a certain genetic exchange between populations, but relatively infrequent. The gene flow of *E. acuminatus* between Patch 1 and Patch 4 was more frequent, whereas the opposite was true for *M. fascifrons* (Figure 4).

### 3.2. Effects of Fragmented Habitats on the Diversity of Leafhoppers

#### 3.2.1. The Isolation Effect at the Landscape Scale Is Detrimental to Leafhopper Community Diversity

The contagion and aggregation indices, used as negative indicators for measuring habitat fragmentation, were on the rise. Their continuous increase over the three-year period indicated a decrease in habitat fragmentation. Additionally, indices including patch density, edge density, and division index—all positive indicators of fragmentation—showed a downward trend (Table 4). The decline of these indicators during this period further confirmed that the habitat fragmentation of leafhoppers was decreasing. Contrary to the increasing trend of genus richness of the leafhopper, the decreasing division index and increasing aggregation index together indicate that the degree of isolation between suitable habitat patches in the study area has decreased. The reduction in the isolation effect may be conducive to the migration and colonization of leafhopper communities, and thereby promote an increase in their richness.

#### 3.2.2. Patch Characteristics and Gene Flow

**Figure 5:**
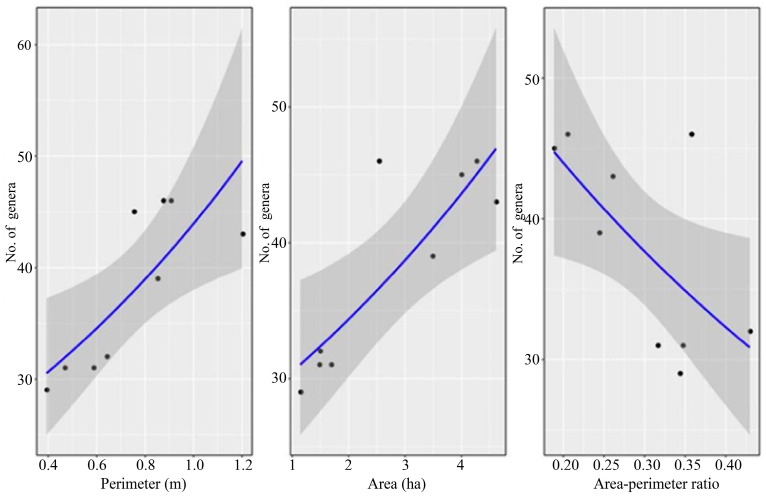
The relationship between generic richness and patch area, perimeter, and perimeter-to-area ratio based on the results of the generalized linear model. Note: The blue solid line represents the fitted regression trend from the model; the gray shaded area denotes the 95% confidence interval of the trend; black dots correspond to observed data points.

Patch area was the best parameter for predicting leafhopper generic richness. As patch area and perimeter increased, leafhopper generic richness also increased, while a larger perimeter-to-area ratio caused generic richness to decrease (Figure 5).

**Figure 6:**
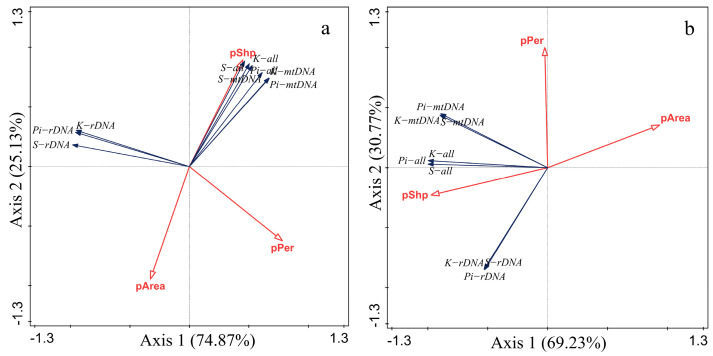
Redundancy analysis between patch characteristics and leafhopper genetic diversity ((**a**). *E. acuminatus*; (**b**). *M. fascifrons*). The definition of each abbreviation in the figure is as follows: (pArea) patch area; (pPer) patch perimeter; (pShp) perimeter-to-area ratio; (Pi−all) total nucleotide diversity; (K−all) total average number of nucleotide differences; (S−all) total number of polymorphic sites; (Pi−rDNA) nuclear gene nucleotide diversity; (K−rDNA) nuclear gene average number of nucleotide differences; (S−rDNA) nuclear gene number of polymorphic sites; (Pi−mtDNA) mitochondrial gene nucleotide diversity; (K−mtDNA) mitochondrial gene average number of nucleotide differences; (S−mtDNA) mitochondrial gene number of polymorphic sites.

The patch perimeter-to-area ratio (pShp) was positively correlated with the nucleotide diversity (Pi), the average number of nucleotide differences (K) and the number of polymorphic sites (S) based on the mitochondrial gene mtDNA and nuclear gene rDNA of the leafhopper, while the patch perimeter pPer and the patch area pArea were negatively correlated with these values (Figure 6). Genetic diversity of the two leafhopper species was proportional to the perimeter-to-area ratio (pShp), indicating that the more irregular the patch shape and the more complex the habitat patch boundary, the greater genetic variation within the patch.

### 3.3. Effects of Plant Composition and Meteorological Factors on Community Structure and Generic Diversity of Leafhoppers

**Figure 7:**
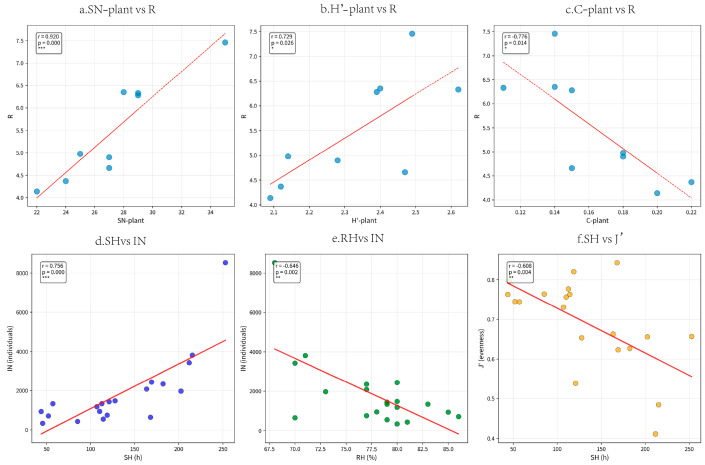
Scatter plots of the correlations between climatic factors/plant diversity and leafhopper diversity, where r represents the correlation coefficient and *p* indicates significance; * 0.01 ≤ *p* < 0.05; ** 0.001 ≤ *p* < 0.05; *** *p* < 0.001). (**a**) Scatter plots of the correlations between plant species richness and Margalef richness index of leafhopper; (**b**) Scatter plots of the correlations between Shannon–Wiener diversity index of plant and Margalef richness index of leafhopper; (**c**) Scatter plots of the correlations between Simpson dominance index of plant and Margalef richness index of leafhopper; (**d**) Scatter plots of the correlations between sunshine hours and number of individuals; (**e**) Scatter plots of the correlations between relative humidity and number of individuals; (**f**) Scatter plots of the correlations between sunshine hours and Pielou evenness index.The definition of each abbreviation in the figure is as follows: (SN−plant) plant species richness; (H′−plant) Shannon–Wiener diversity index of plant; (C−plant) Simpson dominance index of plant; (R) Margalef richness index of leafhopper; (IN) number of individuals; (J′) Pielou evenness index; (RH) relative humidity; (SH) sunshine hours.

Most plant diversity parameters were positively correlated with leafhopper diversity. Overall, the average temperature (T) and sunshine hours (SH) were the main meteorological factors affecting the diversity of leafhoppers. Sunshine hours were strongly positively correlated with the total number of individuals (r = 0.756). Humidity is moderately negatively correlated with the total number of individuals (r = −0.646), meaning that the number of leafhoppers is relatively low in high-humidity environments. Sunshine hours were moderately negatively correlated with the evenness index (r = −0.608), suggesting that leafhoppers were more unevenly distributed when sunshine hours were long. (Figure 7).

**Figure 8:**
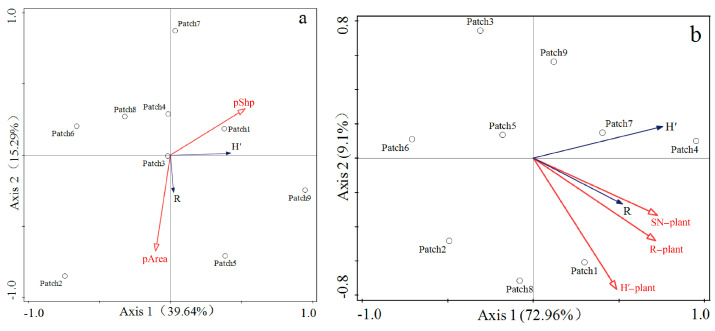
(**a**) Partial redundancy analysis between leafhopper diversity and patch characteristics. (**b**) Partial redundancy analysis between leafhopper diversity and vegetation characteristics. The definition of each abbreviation in the figure is as follows: (H′) Shannon–Wiener diversity index of leafhopper; (R) Margalef richness index of leafhopper. (SN−plant) plant species richness; (H′−plant) Shannon–Wiener diversity index of plant; (R−plant) Margalef richness index of plant; (pShp) perimeter-to-area ratio; (pArea) patch area.

Through partial redundancy analysis (pRDA) with vegetation characteristics and patch characteristics used as control variables, respectively, it was found that the cumulative explanatory power of vegetation characteristics (SN-plant: plant species richness; H′-plant: Shannon–Wiener diversity index of plant; R-plant: Margalef richness index of plant) on leafhopper diversity indices (H′: Shannon–Wiener diversity index of leafhopper; R: Margalef richness index of leafhopper) reached 82.06%, which was far higher than the 54.93% cumulative explanatory power of habitat patch characteristics (pPer: perimeter-to-area ratio; pArea: patch area) on leafhopper diversity (Figure 8). In other words, the impact of vegetation on leafhopper communities in fragmented habitats may be more direct than that of habitat patch fragmentation characteristics.

## 4. Discussion

Changes in the abundance and diversity of leafhoppers, being a phytophagous group, can reflect the health of the ecosystem. Statistical results of the research carried out from 2019 to 2021 showed that a decrease in the habitat isolation effect in the landscape may be accompanied by an increase in the number of leafhopper individuals and their genus richness, which coincided with the decrease in habitat fragmentation in the study area. In previous studies on the correlation between insects and ecological environments, insect species richness and abundance decreased with the destruction of the ecological environment, with the increase in habitat fragmentation, and with the reduction in suitable habitat area [12,47,48]. Habitat fragmentation has a negative impact on the abundance and species richness of herbivorous insects [49,50]. This study supports these conclusions, showing that reduced habitat fragmentation has a positive effect on leafhopper richness. The reduction in habitat fragmentation serves as a means to control karst rocky desertification and plays a positive role in regional biodiversity conservation and ecosystem health [51].

Long sunshine hours and high temperatures tend to make certain thermophilic leafhoppers more active, thereby leading to an increase in the number of captured leafhoppers and resulting in their uneven distribution. In the study area, the number of leafhopper individuals reached its peak in July and August, while significantly lower population abundances were observed in other months, which was consistent with the results of previous studies [52,53,54]. Higher summer temperatures likely facilitate leafhopper development and reproduction, leading to high abundances [52,55,56]. Statistical analyses revealed a significant negative effect of air humidity on leafhopper generic richness, indicating that persistent rainfall and high humidity may inhibit the development and fecundity of certain leafhopper species, and may cause higher mortality.

Although the study found that the increase in patch area has a positive effect on leafhopper diversity, the results of partial redundancy analysis (pRDA) showed that the explanatory power of vegetation characteristics for leafhopper diversity at the patch scale was higher than that of habitat patch characteristics. In other words, the diversity of leafhopper communities in fragmented habitats may be simultaneously affected by the complex effects of multiple factors, including vegetation and habitat microclimate.

Different landscape parameters may exert distinctly different impacts on biodiversity. For instance, a high perimeter-to-area ratio had a negative impact on leafhopper generic richness; however, in the study area, patches with a small area but a large perimeter-to-area ratio exhibited higher genetic diversity, which is somewhat inconsistent with our expectations. Habitat fragmentation mainly affects the gene flow between populations by increasing isolation and reducing the number of effective populations [57]. In this study, the distance to the nearest suitable patch had no significant impact on leafhopper generic richness. This may be because the distance between the sampled patches and the nearest suitable patches was not sufficiently large; meanwhile, croplands around the patches may provide temporary habitats for some leafhopper species [58]. The nucleotide diversity (Pi) of the three patches was consistent with the order of the patch perimeter-to-area ratio. Patches with large perimeter-to-area ratios indicate greater edge heterogeneity (e.g., diverse microhabitats, complex vegetation structure), providing these two leafhopper species (i.e., *E. acuminatus* and *M. fascifrons*) with a wider range of resources and environmental conditions. This heterogeneity facilitates more frequent gene exchange and higher genetic diversity among individuals of these two species. In addition to patch characteristics (e.g., perimeter-to-area ratio), intrinsic biological traits of the two leafhopper species studied—such as migration ability, population size, and life cycle—also influence inter-patch gene flow [59].

Leafhopper community structure and diversity were significantly influenced by plant species composition. Rowe and Holland (2013) suggested that higher plant richness leads to an increase in leafhopper diversity and richness, which aligns with our study results [60]. We found that some leafhopper generic richness increased with the abundance of specific plant species. For example, *Dikraneura* spp. and *Artemisia argyi* exhibited a significant positive correlation, indicating that certain leafhopper species may have a strong dependence on specific host plants. According to Yin (1992), more than half of leafhopper species are polyphagous, nearly half are oligophagous, and some feed on only one host plant (monophagous) [61,62]. This dependence sometimes leads to the ‘host specificity’ of leafhoppers (i.e., exclusive dependence on one or a few host plant species), which has important implications for their survival and distribution. The generic richness of leafhoppers (R) was positively influenced by plant species richness (R-plant) and diversity, but negatively correlated with plant dominance index (C-plant). Current research on soil impacts on leafhoppers primarily focuses on land use types (e.g., cropland, forest, urbanization) or intensity, with almost no studies addressing the effects of soil physicochemical properties [63]. This study found that soil physicochemical properties had a weak direct effect on leafhopper diversity.

## 5. Conclusions

Our results from multi-year leafhopper sampling in light-to-moderate rocky desertification zones demonstrated that leafhopper generic diversity in fragmented habitats of the Guizhou karst region was governed by a multi-tiered driving mechanism: “vegetation characteristics as the core, habitat patch attributes as regulators, and climatic factors as synergists”. Specifically, partial redundancy analysis (pRDA) revealed that patch-scale vegetation characteristics exerted significantly greater explanatory power over leafhopper diversity than habitat patch attributes, thus confirming that vegetation characteristics were the most critical driver of leafhopper generic diversity.

While increasing patch area had a positive impact on leafhopper diversity, the patch perimeter–area ratio exhibited a “dual role”: it exerted a negative effect on leafhopper generic richness, yet a positive effect on the genetic diversity (Pi) of the two focal target leafhopper species (*E. acuminatus* and *M. fascifrons*).

This finding highlighted that vegetation should be the centerpiece of habitat restoration in the karst region: priority should be accorded to the protection of native plant communities to enhance vegetation diversity, thereby providing a stable resource pool for phytophagous insects such as leafhoppers. While preserving large continuous habitats as “core shelters”, small heterogeneous habitat patches should be connected via ecological corridors to maximize their role in sustaining ecosystem diversity.

## Figures and Tables

**Table 1:** Composition of dominant species, common species, and rare species of leafhoppers in different years.

Years	Number and Proportion of			Number and Proportion of Individuals of		
	Dominant Genera	Common Genera	Rare Genera	Dominant Genera	Common Genera	Rare Genera
2019	65 (84.42%)	7 (9.09%)	5 (6.49%)	6099 (72.23%)	1184 (14.02%)	1161 (13.75%)
2020	65 (78.31%)	14 (16.87%)	4 (4.82%)	5438 (59.56%)	2604 (28.52%)	1089 (11.93%)
2021	63 (75.90%)	16 (19.28%)	4 (4.82%)	4955 (52.72%)	3449 (36.70%)	955 (10.16%)
2022	64 (79.01%)	13 (16.05%)	4 (4.82%)	3334 (34.35%)	5130 (52.85%)	1243 (12.81%)

**Table 2:** Differences in community structure and dominance of leafhoppers in different patches in 2022.

Patch	Number and Proportion of			Number and Proportion of Individuals of		
	Dominant Genera	Common Genera	Rare Genera	Dominant Genera	Common Genera	Rare Genera
Patch 1	4 (8.70%)	10 (21.74%)	32 (69.57%)	823 (67.07%)	282 (22.98%)	122 (9.94%)
Patch 2	4 (8.70%)	9 (19.57%)	33 (71.74%)	798 (61.43%)	357 (27.48%)	144 (11.09%)
Patch 3	6 (15.38%)	6 (15.38%)	27 (69.23%)	1725 (84.11%)	217 (10.58%)	109 (5.31%)
Patch 4	7 (15.56%)	13 (28.89%)	25 (55.56%)	227 (54.70%)	141 (33.98%)	47 (11.33%)
Patch 5	6 (13.95%)	9 (20.93%)	28 (65.12%)	776 (64.77%)	314 (26.21%)	108 (9.02%)
Patch 6	6 (19.35%)	5 (16.13%)	20 (64.52%)	1201 (86.28%)	116 (8.33%)	75 (5.39%)
Patch 7	5 (17.24%)	9 (31.03%)	15 (51.72%)	221 (73.18%)	62 (20.53%)	19 (6.29%)
Patch 8	4 (12.90%)	9 (29.03%)	18 (58.06%)	466 (74.56%)	124 (19.84%)	35 (5.60%)
Patch 9	4 (12.50%)	13 (40.63%)	15 (46.88%)	799 (66.14%)	344 (28.48%)	65 (5.38%)

**Table 3:** Genetic diversity analysis of different patch populations of *E. acuminatus* and *M. fascifrons* calculated based on mtDNA and rDNA.

Species	Gene	Patches	Nucleotide Diversity	Average Number of Nucleotide Differences	Number of Polymorphic Sites
*E. acuminatus*	mtDNA	Patch1	0.08258	127.333	185
		Patch4	0.05793	89.333	132
		Patch9	0.08344	128.667	193
	rDNA	Patch1	0.00548	7.667	11
		Patch4	0.01097	15.333	23
		Patch9	0.01144	16.000	22
	mtDNA + rDNA	Patch1	0.04592	135.000	196
		Patch4	0.03560	104.667	155
		Patch9	0.04921	144.667	215
*M. fascifrons*	mtDNA	Patch1	0.17279	268.000	385
		Patch4	0.03954	61.333	91
		Patch9	0.12035	186.667	273
	rDNA	Patch1	0.00285	4.000	6
		Patch4	0.00523	7.333	10
		Patch9	0.06177	86.667	130
	mtDNA + rDNA	Patch1	0.09208	272.000	391
		Patch4	0.02325	68.667	101
		Patch9	0.09253	273.333	403

**Table 4:** Changes in the fragmentation index of the study area from 2019 to 2021.

Years	Patch Density	Edge Density	Contagion Index	Division Index	Aggregation Index
2019	0.2280	10.9585	53.7434	0.7536	83.4282
2020	0.2238	10.8618	54.3878	0.7500	83.5698
2021	0.2205	10.6159	55.1561	0.7471	83.9374

## Data Availability

The original contributions presented in the study are included in the article. Further inquiries can be directed to the corresponding author.
